# Pharmacological enhancement of mGlu1 metabotropic glutamate receptors causes a prolonged symptomatic benefit in a mouse model of spinocerebellar ataxia type 1

**DOI:** 10.1186/1756-6606-6-48

**Published:** 2013-11-19

**Authors:** Serena Notartomaso, Cristina Zappulla, Francesca Biagioni, Milena Cannella, Domenico Bucci, Giada Mascio, Pamela Scarselli, Francesco Fazio, Filippo Weisz, Luana Lionetto, Maurizio Simmaco, Roberto Gradini, Giuseppe Battaglia, Michele Signore, Aldamaria Puliti, Ferdinando Nicoletti

**Affiliations:** 1 I.R.C.C.S. Neuromed, Pozzilli, Italy; 2 Department of Physiology and Pharmacology, University “Sapienza”, Piazzale Aldo Moro, 5, 00185 Rome, Italy; 3 Department of Neuroscience, Mental Health and Sensory Organs, Advanced Molecular Diagnostics, Azienda Ospedale S. Andrea, Rome, Italy; 4 Department of Experimental Medicine, University “Sapienza”, Rome, Italy; 5 Department of Hematology, Oncology and Molecular Medicine, Istituto Superiore di Sanità, Rome, Italy; 6 Molecular Genetics and Cytogenetics Unit, Gaslini Institute, Genoa, Italy

**Keywords:** mGlu1 receptor, Ro0711401, mGlu5 receptor, VU0360172, JNJ16259685, Spinocerebellar ataxia type 1, Purkinje cell, Motor coordination

## Abstract

**Background:**

Spinocerebellar ataxia type 1 (SCA1) is a genetic disorder characterized by severe ataxia associated with progressive loss of cerebellar Purkinje cells. The mGlu1 metabotropic glutamate receptor plays a key role in mechanisms of activity-dependent synaptic plasticity in the cerebellum, and its dysfunction is linked to the pathophysiology of motor symptoms associated with SCA1. We used SCA1 heterozygous transgenic mice (Q154/Q2) as a model for testing the hypothesis that drugs that enhance mGlu1 receptor function may be good candidates for the medical treatment of SCA1.

**Results:**

Symptomatic 30-week old SCA1 mice showed reduced mGlu1 receptor mRNA and protein levels in the cerebellum. Interestingly, these mice also showed an intense expression of mGlu5 receptors in cerebellar Purkinje cells, which normally lack these receptors. Systemic treatment of SCA1 mice with the mGlu1 receptor positive allosteric modulator (PAM), Ro0711401 (10 mg/kg, s.c.), caused a prolonged improvement of motor performance on the rotarod and the paw-print tests. A single injection of Ro0711401 improved motor symptoms for several days, and no tolerance developed to the drug. In contrast, the mGlu5 receptor PAM, VU0360172 (10 mg/kg, s.c.), caused only a short-lasting improvement of motor symptoms, whereas the mGlu1 receptor antagonist, JNJ16259685 (2.5 mg/kg, i.p.), further impaired motor performance in SCA1 mice. The prolonged symptomatic benefit caused by Ro0711401 outlasted the time of drug clearance from the cerebellum, and was associated with neuroadaptive changes in the cerebellum, such as a striking reduction of the ectopically expressed mGlu5 receptors in Purkinje cells, increases in levels of total and Ser880-phosphorylated GluA2 subunit of AMPA receptors, and changes in the length of spines in the distal dendrites of Purkinje cells.

**Conclusions:**

These data demonstrate that pharmacological enhancement of mGlu1 receptors causes a robust and sustained motor improvement in SCA1 mice, and lay the groundwork for the development of mGlu1 receptor PAMs as novel “cerebellum-specific”, effective, and safe symptomatic drugs for the treatment of SCA1 in humans.

## Background

Spinocerebellar ataxia type-1 (SCA1) is an inherited neurological disease caused by expansion of an unstable CAG repeat in the ataxin-1 (ATXN1) gene, which leads to progressive degeneration of Purkinje cells in the cerebellar cortex. SCA1 patients show loss of coordination of the limbs and trunk, unstable gait, dysarthric speech, and nystagmus, as well as extrapyramidal and dysautonomic symptoms and cognitive dysfunction [1,2]. Several mouse models have been generated over the years in an attempt to shed light on ATXN1 function and its role in the pathogenesis of SCA1. These mouse models recapitulate the histopathological and behavioral hallmarks of SCA1 patients. For example, SCA1^154Q/2Q^ mice show progressive degeneration of Purkinje cells, ataxia, muscle wasting, severe kyphosis, and premature death between 35 and 45 weeks of age [3]. A current theory on the pathogenesis of SCA1 is that polyglutamine expansion disrupts the physiological interaction between ATXN1 and nuclear proteins regulating gene transcription and RNA processing, such as Capicua, retinoic acid orphan receptor-α (RORα), and RBM17 [4]. None of these mechanisms can be easily targeted by pharmacological interventions, and there is currently no treatment for SCA1 and other types of spinocerebellar ataxia.

Although nuclear mechanisms lie at the core of the disorder, type-1 metabotropic glutamate receptors (mGlu1 receptors) are tightly linked to the pathophysiology of SCA1. mGlu1 receptors are coupled to Gq/11 and their activation stimulates the hydrolysis of phosphatidyinositol-4,5-bisphosphate with ensuing formation of inositol-1,4,5-trisphosphate (InsP_3_) and diacylglycerol (DAG). InsP_3_ stimulates Ca^2+^ release from intracellular stores, whereas DAG activates protein kinase C (PKC) [5]. The cognate mGlu5 receptor is also coupled to inositol phospholipid hydrolysis but with diversity of Ca^2+^ signaling. In recombinant cells, activation of mGlu1α receptors induces an increase in intracellular free Ca^2+^ consisting of an initial transient peak followed by a steady plateau phase, whereas activation of mGlu5a receptors induces intracellular Ca^2+^ oscillations [6]. Another difference between mGlu1 and mGlu5 receptors is the developmental pattern of expression. mGlu1 receptors predominate in cerebellar Purkinje cells [7], where their expression levels increase with age [8]. In contrast, expression and function of mGlu5 receptors is high in the first 2 weeks of postnatal life and declines afterwards [9].

mGlu1 receptors are essential for the induction of long-term depression (LTD) at both parallel fiber and climbing fiber-Purkinje cell synapses, a form of activity-dependent synaptic plasticity underlying cerebellar motor learning and vestibule-ocular reflex adaptation [10-14]. Mice with genetic deletion of mGlu1 receptors show a profound defect of cerebellar LTD, motor coordination and conditioned eyeblink reflex [14-17], and all these defects are corrected by selective re-introduction of mGlu1 receptors in Purkinje cells [18].

Interestingly, a defective expression/activity of mGlu1 receptors or downstream signaling molecules is found in genetic mouse models of ataxia [19-21], and autoantibody directed against mGlu1 receptors are occasionally present in patients with paraneoplastic ataxia associated with Hodgkin’s lymphoma [22].

The link between SCA1 and mGlu1 receptors is supported by the following observations: (i) one of the earliest pathological events in SCA1 B05/+ mice is the presence of cytoplasmic vacuoles containing mGlu1 receptors in cerebellar Purkinje cells [23]; (ii) in conditional SCA1[82Q] mice, motor recover after interruption of transgene expression is associated with reappearance of mGlu1 receptors at parallel fiber-Purkinje cell synapses [24]; (iii) homozygous *staggerer* mutant mice, which are considered as an “extreme” mouse model of SCA1, lack mGlu1 receptor-mediated slow synaptic transmission at parallel fiber-Purkinje cell synapses and show low expression levels of mGlu1 receptors in the cerebellum [21]; and (iv) expression of genes encoding for mGlu1 receptor signaling proteins, such as Homer-3 and type-1 InsP_3_ receptors, is altered in cerebellar Purkinje cells of SCA1 B05 mice [25,26]. These studies laid the groundwork for targeting mGlu1 receptors in the treatment of SCA1. New subtype-selective drugs that amplify mGlu1 receptor function by interacting with a site different from the glutamate recognition site are available [27-29]. These positive allosteric modulators (PAMs) or “enhancers” are highly promising from a therapeutical standpoint because they exclusively recruit mGlu receptors that are activated by endogenous glutamate, thus acting in an “activity-dependent” manner. We now report that, systemic administration of a selective mGlu1 receptor PAM to SCA1 mutant mice, causes long-lasting improvements in motor symptoms associated with adaptive changes in cerebellar neuroplasticity.

## Results

### Changes in the expression of mGlu1α and mGlu5 receptors in Purkinje cells of symptomatic SCA1 mice

**Figure 1:**
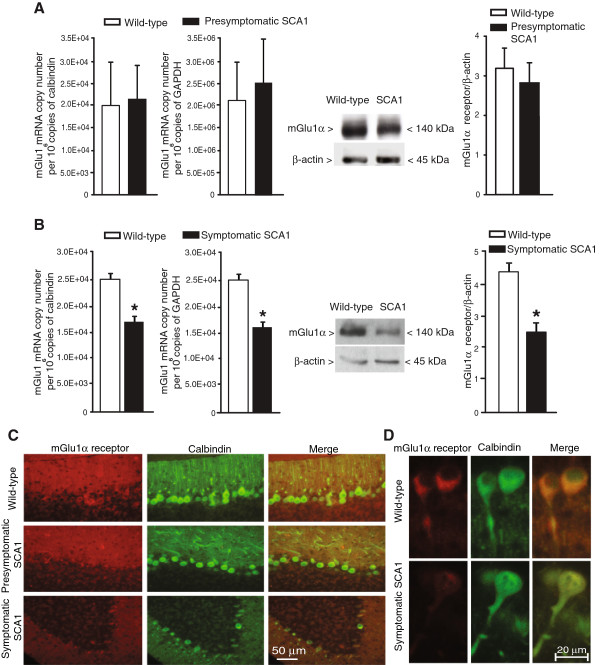
**Reduced mGlu1 receptor mRNA and protein levels in the cerebellum of symptomatic SCA1 mice.** mGlu1 receptor mRNA and mGlu1α receptor protein levels in the cerebellum of presymptomatic and symptomatic SCA1 mice (and their age-matched wild-type littermates) are shown in **(A)** and **(B)**, respectively. Values are means ± S.E.M. of 3–4 mice per group. *p < 0.05 (Student’s t test) vs. the corresponding wild-type mice; t values = 5.3 (mRNA values normalized to GAPDH); 6.5 (mRNA values normalized to calbindin); and 7.075 (densitometric analysis of immunoblots). Immunohistochemical analysis of mGlu1α receptors and calbindin in the cerebellum of SCA1 mice and wild-type littermates are shown in **(C)** and **(D)**.

We measured mGlu1 receptor mRNA and mGlu1α receptor protein levels by real-time PCR and immunoblotting, respectively. The mGlu1α antibody detected a major band at 140 kDa corresponding to the deduced molecular size of receptor monomers. Labeling was highly specific because the band disappeared in the cerebellum of mGlu1-deficient *crv4* mice [17] (not shown). mGlu1α mRNA and protein levels in the cerebellum did not differ between 4-week old presymptomatic SCA1 mice and their age-matched wild-type littermates (Figure 1A). In contrast, 30-week old symptomatic SCA1 mice showed large reductions in mGlu1 receptor mRNA levels, which were equally seen when data were normalized to both GAPDH and calbindin mRNA levels (Figure 1B). mGlu1α receptor protein levels were also reduced by about 50% in the cerebellum of symptomatic SCA1 mice, at least when expression data were normalized to β-actin levels (Figure 1B). Immunohistochemical analysis showed a reduced intensity of mGlu1α receptor staining in symptomatic SCA1 mice, which was particularly evident in the dendritic arborization of Purkinje cells (Figure 1C). High magnification analysis showed that mGlu1α receptor protein expression was also reduced at least in some Purkinje cells that appeared morphologically intact and were regularly stained with anti-calbindin antibody (Figure 1D). Taken together, these data indicated that a loss of mGlu1α receptors in Purkinje cells was associated with the pathological phenotype of SCA1 mice.

**Figure 2:**
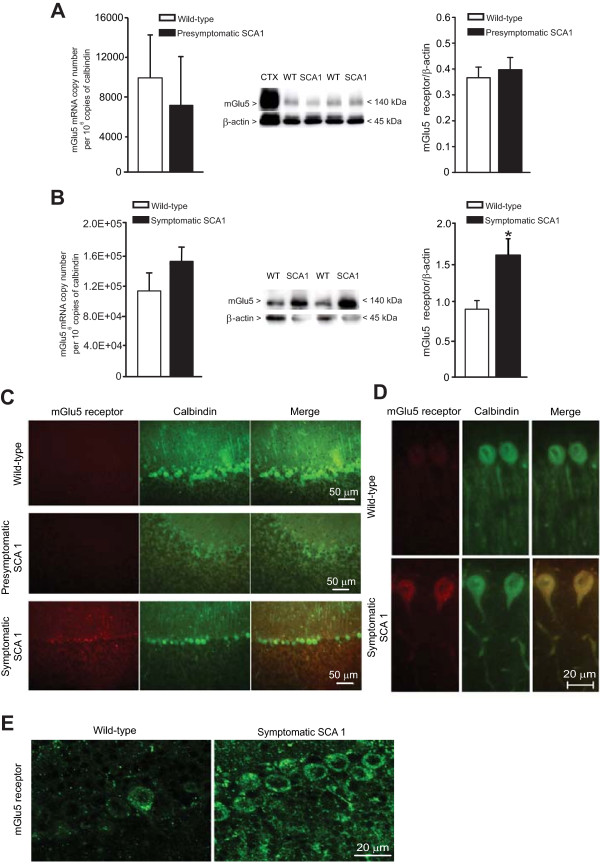
**Appearance of mGlu5 receptors in Purkinje cells of symptomatic SCA1 mice.** mGlu5 receptor mRNA and protein levels in the cerebellum of presymptomatic and symptomatic SCA1 mice (and their age-matched wild-type littermates) are shown in **(A)** and **(B)**, respectively. Values are means ± S.E.M. of 4 mice per group. *p < 0.05 (Student’s t test) *vs.* the corresponding wild-type mice. t = 10 (densitometric analysis of immunoblots). Immunohistochemical analysis of mGlu5 receptors in the cerebellum of SCA1 mice and wild-type littermates are shown in **(C)** and **(D)**. Confocal microscopy analysis of mGlu5 receptors in Purkinje cells of wild-type and symptomatic SCA1 mice is shown in **(E)**. Note the somatic and perisomatic immunostaining which excludes the cell nucleus.

We extended the analysis to mGlu5 receptors, which are normally expressed at low levels in the adult cerebellum and are nearly absent in Purkinje cells (see Figure 2C). The band at 140 kDa corresponding to mGlu5 receptor monomers was absent in the cerebral cortex of mGlu5 receptor knockout mice (not shown) confirming the specificity of the antibody. Expression of mGlu5 receptors was unaffected in the cerebellum of presymptomatic SCA1 mice (Figure 2A). In contrast, we found a large increase of mGlu5 receptor protein levels and a trend to an increase in the transcript of mGlu5 receptors in symptomatic SCA1 mice (Figure 2B). The induction of mGlu5 receptor protein was fully confirmed by immunohistochemical analysis, where Purkinje cells from only symptomatic SCA1 mice were stained by anti-mGlu5 receptor antibodies. Both cell bodies and dendrites of Purkinje cells expressed mGlu5 receptors in symptomatic SCA1 mice (Figure 2C-E). Confocal microscopy analysis showed a perisomatic localization of mGlu5 receptors in Purkinje cells of SCA1 mice (Figure 2E).

### Pharmacological enhancement of mGlu1 receptors caused a long-lasting improvement of motor coordination in SCA1 mice

**Figure 3:**
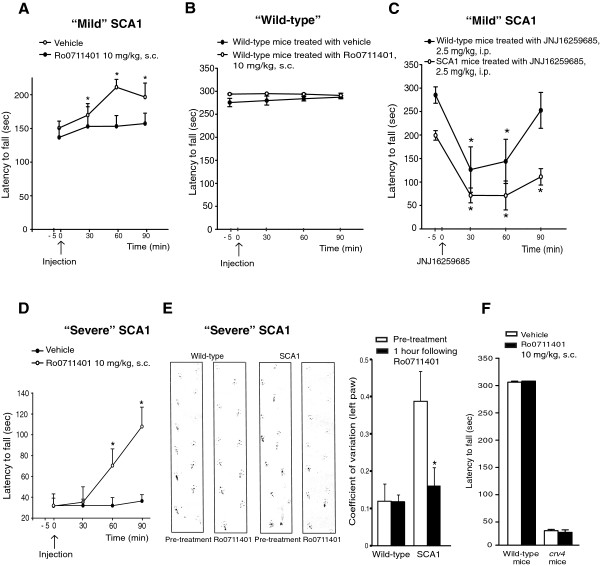
**Acute treatment with the mGlu1 receptor PAM, Ro0711401, improves motor performance in symptomatic SCA1 mice.** Effect of Ro0711401 on the rotarod in symptomatic SCA1 mice with mild motor impairment **(A)**; values are means ± S.E.M. of 4 mice per group. *p < 0.05 vs. corresponding values at time 0; Two-way ANOVA for repeated measures + Fisher’s LSD: F_(3,21)_ = 7.621 and F_(1,21)_ = 10.176 for time and treatment, respectively; and in wild-type littermates **(B)**; data are means ± S.E.M. of 7 mice per group. Lack of effect of JNJ16259685 in SCA1 mice with mild motor impairment and wild-type **(C)**, where data are means ± S.E.M. of 4 mice per group; *p < 0.05 *vs.* respective values at time 0. Two-way ANOVA for repeated measures + Fisher’s LSD; F_(3,18)_ = 34.38 and F_(1,18)_ = 7.24 for time and treatment, respectively. Effect of Ro0711401 in symptomatic SCA1 mice with severe motor impairment **(D)**; values are means ± S.E.M. of 5 mice per group. *p < 0.05 vs. corresponding values at time 0; Two-way ANOVA for repeated measures + Fisher’s LSD; F_(3,12)_ = 11.177 and F_(4,39)_ = 9.982 for time and time x treatment, respectively. Paw print test in SCA1 mice with severe motor impairment and wild-type acutely treated with Ro0711401 **(E)**. Data were quantified by calculating the coefficient of variation of 5 consecutive strides with the left paw of each mouse. Values are means ± S.E.M. of 9 mice. *p < 0.05 (One-Way ANOVA plus Fisher’s PLSD) *vs.* SCA1 mice before the treatment. Representative traces were carried out 5 min prior and 60 min after drug injection. Motor performance on the rotarod of mGlu1-deficient *crv4* mice and wild-type mice 1 hour following s.c. injection of vehicle or Ro0711401 **(F)**. Values are means + S.E.M. of 4 mice per group.

Thirty-week old SCA1 mice showed a reduced motor performance on the rotarod, and signs of ataxia in the paw print test. However, the extent of motor impairment in these mice was variable, with some of them showing a mild motor impairment and other a severe impairment at the rotarod. Motor performance in SCA1 mice with mild impairment was slightly but significantly improved by a single systemic administration with the mGlu1 receptor PAM, Ro0711401 (10 mg/kg, s.c.) at all times from 30 to 90 min post-injection (Figure 3A). Injection of Ro0711401 had no effect on motor performance in wild-type littermates (Figure 3B). In contrast, single injection of the mGlu1 receptor negative allosteric modulator (NAM), JNJ16259685 (2.5 mg/kg, i.p.), markedly reduced motor performance both in wild-type and 30-week old SCA1 with mild motor impairment at 30 and 60 min post-injection (Figure 3C). In mice with severe impairment of motor performance (latency to fall < 100 sec on the rotarod), a single injection with Ro0711401 caused a large improvement of motor coordination, which was visible at 60 and 90 min post injection (Figure 3D,E). To exclude the possibility that Ro0711401 increased motor performance by an off-target effect, we tested the compound in mGlu1-deficient *crv4* mice, which display severe ataxia [17]. As opposed to what observed in SCA1 mice, a single injection of Ro0711401 (10 mg/kg, s.c.) did not improve motor performance on the rotarod in *crv4* mice (Figure 3F).

**Figure 4:**
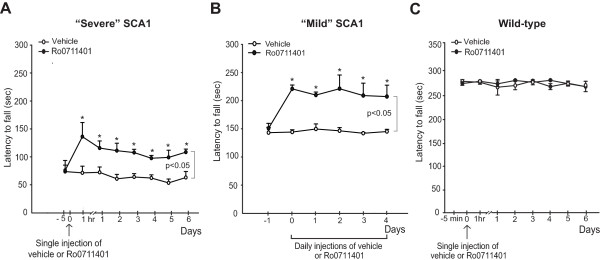
**Pharmacological enhancement of mGlu1 receptors causes a prolonged symptomatic benefit in SCA1 mice.** Motor performance on the rotarod in SCA1 mice with severe motor impairment at different times following a single injection of vehicle or Ro0711401 is shown in **(A)**, where values are means ± S.E.M. of 6 mice per group. *p < 0.05 *vs.* the corresponding values at time 0; Two-way ANOVA for repeated measures + Fisher’s LSD; F_(7,35)_ = 2.48, F_(1,10)_ = 21.003, and F_(7,35)_ = 2.321 for time, treatment, and time x treatment, respectively. Motor performance in SCA1 mice with mild impairment treated daily with Ro0711401 is shown in **(B)**, where values are means ± S.E.M. of 6 mice per group. *p < 0.05 *vs.* the corresponding pre-treatment values; Two-way ANOVA for repeated measures + Fisher’s LSD; F_(5,15)_ = 4.248, F_(1,11)_ = 37.408 and F_(5,15)_ = 6.078 for time, treatment and time x treatment, respectively. The lack of motor effect of single injection of Ro0711401 in wild-type littermates is shown in **(C)**, where values are means ± S.E.M. of 4 mice per group.

We next examined the temporal profile of response to a single injection of Ro0711401 in SCA1 mice by assessing motor performance on the rotarod every day for 6 days. Surprisingly, a single administration of Ro0711401 caused a long-lasting improvement in motor performance, which was maintained to the same extent at least for 6 days (Figure 4A). Again, the drug had no effect in wild-type littermates (Figure 4C). We also tested motor performance in response to daily administrations of Ro0711401 (10 mg/kg, s.c., for 6 days) showing no development of tolerance to the motor-improving effect of the drug (Figure 4B).

**Figure 5:**
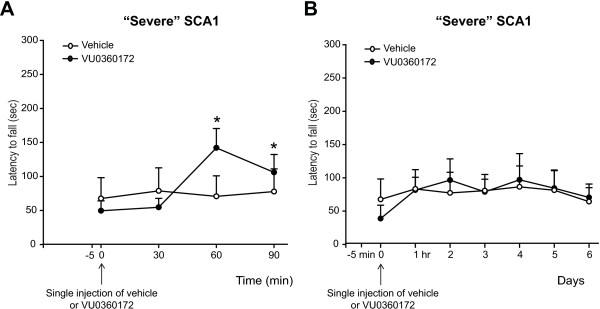
**A Single injection of the mGlu5 receptor PAM, VU0360172, causes a short-lasting improvement of motor performance in symptomatic SCA1 mice.** Motor performance on the rotarod in SCA1 mice with severe motor impairment treated with a single injection of VU0360172 is shown. In **(A)**, values are means ± S.E.M. of 5 mice per group. *p < 0.05 *vs.* the corresponding values at time 0; Two-way ANOVA for repeated measures + Fisher’s LSD; F_(3,12)_ = 5.921, F_(3,12)_ = 5.629 for time and time x treatment, respectively. In **(B)**, values are means ± S.E.M. of 4 mice per group.

We also examined whether pharmacological enhancement of mGlu5 receptors could also be beneficial in SCA1 mice using the selective mGlu5 receptor PAM, VU0360172 (10 mg/kg, s.c.). A single administration of VU0360172 transiently improved motor symptoms in SCA1 mice at 60 and 90 min post-injection (Figure 5A). As opposed to Ro0711401, VU0360172 had no enduring effects on motor behavior, and there was no significant difference between mice treated with VU0360172 and those treated with vehicle when motor performance was assessed at 1 to 6 days post-injection (Figure 5B).

### Potential mechanisms underlying the long-lasting improvement in motor symptoms caused by Ro0711401 in SCA1 mice

a) **Measurements of Ro0711401 levels in the cerebellum**

We first examined whether the long-lasting effects of Ro0711401 in symptomatic SCA1 mice were due to a slow clearance of the drug from the cerebellum. Drug levels were detected by HPLC/MS-MS at 30–120 min after a single injection of Ro0711401 (10 mg/kg, s.c.), confirming that the drug crosses the blood–brain barrier. However, levels became undetectable at 24 hours post-injection, excluding that the enduring increase in motor performance was due to the maintenance of Ro0711401 in cerebellar tissue (Table 1).

**Table 1:** **Measurements of drug levels by HPLC/MS-MS in the cerebellum of symptomatic SCA1 mice at 30**–**120 min after a single injection of Ro0711401 (10 mg/kg, s.c.)**

**Time after injection**	**Cerebellar Ro0711401 levels**
	**(pg/mg tissue)**
30 min	211 ± 20
60 min	135 ± 10
90 min	100 ± 9
120 min	100 ± 40
240 min	80 ± 30
24 hours	N.D.

Values are means ± S.E.M. of 3 animals per time point. N.D. = not detectable.

b) **Changes in mGlu1α and mGlu5 receptor expression in response to a single injection of Ro0711401**

**Figure 6:**
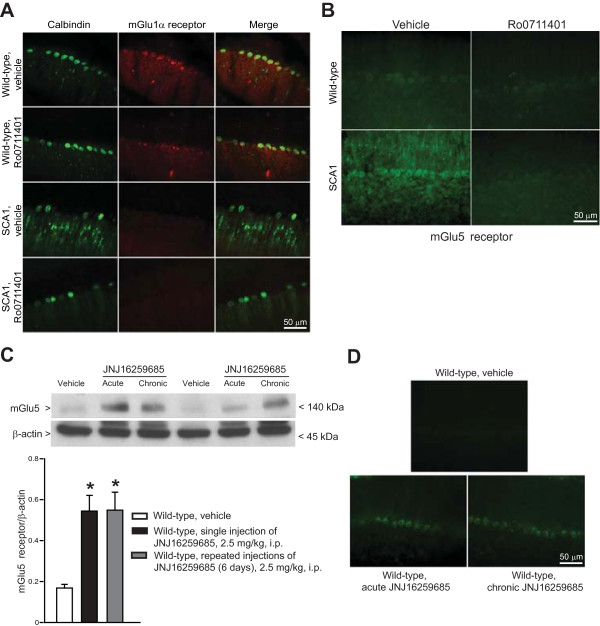
**Changes in the expression of mGlu1α or mGlu5 receptors following treatments with Ro0711401 or JNJ16259685.** Immunohistochemical analysis of mGlu1α and mGlu5 receptors in cerebellar serial sections of symptomatic SCA1 mice 6 days following a single s.c. injection of vehicle or Ro0711401 (10 mg/kg) is shown in **(A)** and **(B)**, respectively. Note the disappearance of mGlu5 receptors from Purkinje cells after treatment with Ro0711401. Immunoblot analysis of mGlu5 receptors in wild-type mice treated acutely or repeatedly with JNJ16259685 (2.5 mg/kg) is shown in **(C)**. Control mice were treated daily i.p. with vehicle for 6 days. The group of mice indicated as “single injection of JNJ16259685” received a 6-day treatment of vehicle followed by a single administration of JNJ16259685. The third group of mice received daily injections of JNJ16259685 for 6 days. A representative immunoblot of mGlu5 receptor is shown. Densitometric values are means ± S.E.M. of 5 mice per group. *p < 0.05 *vs.* mice treated with vehicle (One-way ANOVA + Fisher’s LSD). F_(2,12)_ = 13.124. Immunohistochemical analysis of mGlu5 receptors in the cerebellum of the same mice is shown in **(D)**.

We next examined whether a single injection with Ro0711401 could induce adaptive changes in the expression of mGlu1α and mGlu5 receptors in the cerebellum. Immunohistochemical analysis carried out at 6 days post-injection confirmed the reduced expression of mGlu1α receptors in the cerebellum of SCA1 mice, which was unaltered by drug treatment (Figure 6A). In contrast, single injection of Ro0711401 abolished the increase in the expression of mGlu5 receptors seen in the Purkinje cells of symptomatic SCA1 mice (Figure 6B). These data raised the interesting possibility that mGlu1 receptors negatively regulate the expression of mGlu5 receptors in cerebellar Purkinje cells. To further examine this possibility, we measured mGlu5 receptors in wild-type mice treated with the mGlu1 receptor NAM, JNJ16259685. Mice were killed 6 days following a single injection (“acute treatment”) or after 6 daily injections (“chronic treatment”) with JNJ16259685 (2.5 mg/kg, i.p.). Under both paradigms of administration, pharmacological blockade of mGlu1 receptors caused large increases in mGlu5 receptor protein levels in the cerebellum of wild-type mice (Figure 6C). Immunohistochemical analysis showed a strong mGlu5 receptor staining in Purkinje cells of mice treated with JNJ16259685 (Figure 6D).

c) **A single injection of Ro0711401 did not change the distribution profile of vGluT2 immunostaining in the cerebellar cortex of SCA1 mice**

**Figure 7:**
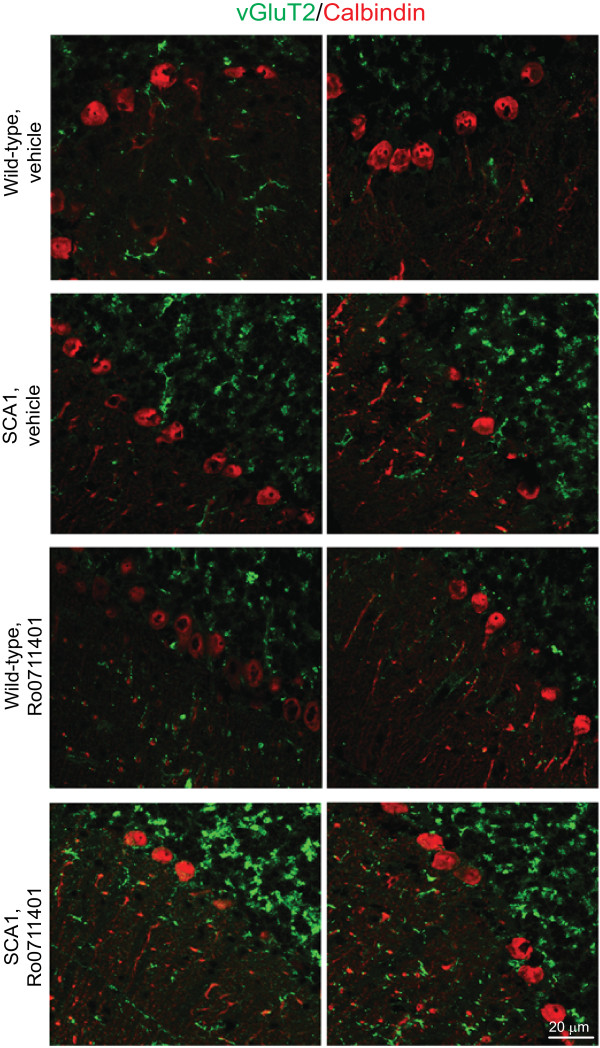
**Ro0711401 treatment does not change the distribution profile of vGluT2 immunostaining in the cerebellum of wild-type and symptomatic SCA1 mice.** Wild-type and SCA1 mice (n = 3 per group) received a single s.c. injection of vehicle or Ro0711401 (10 mg/kg), and were killed after 6 days. Representative sections from 2 individual mice per group are shown. Note the increased vGluT2 immunostaining in the cerebellar granular layer of SCA1 mice.

Knowing that deletion of mGlu1 receptors causes an impairment of developmental elimination of redundant climbing fibers to Purkinje cell synapses [30], we examined the distribution pattern of putative vGluT-2-immunoreactive climbing fibers in the cerebellar cortex of wild-type or SCA1 mice 6 days following an acute injection of vehicle or Ro0711401. SCA1 mice showed a large increase in vGluT-2 immunoreactivity in the granular layer of the cerebellar cortex (Figure 7), which is suggestive of an increased number of *en passant* climbing fibers projecting to Purkinje cells. This was consistent with the decreased pruning of climbing fiber terminals found in other mouse models of SCA1 [31]. Treatment with Ro0711401 did not change the distribution profile of vGluT-2 immunoreactivity in both wild-type and SCA1 mice (Figure 7).

d) **A single injection of Ro0711401 caused adaptive changes typically associated with activity-dependent synaptic plasticity in the cerebellum**

**Figure 8:**
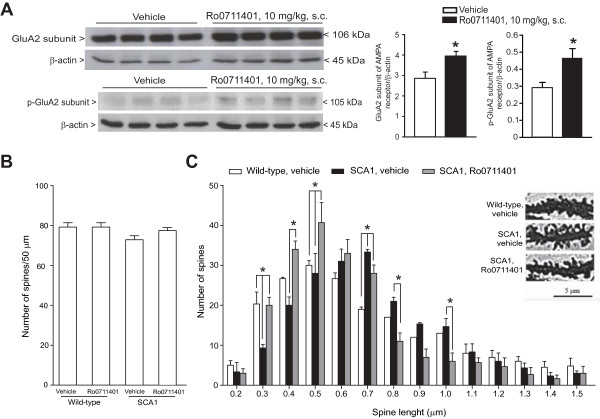
**Neuroadaptive changes caused by a single injection of Ro0711401 in the cerebellum of SCA1 mice.** Levels of total and Ser880-phosphorylated GluA2 subunit of AMPA receptors in the cerebellum of symptomatic SCA1 mice 6 days following a single injection of vehicle or Ro0711401 (10 mg/kg, s.c.) is shown in **(A)**. Densitometric values are means ± S.E.M. of 4–5 mice per group. *p < 0.05 *vs.* mice treated with vehicle (Student’s t test). t = - 3.192 (total GluA2) and 4.311 (p-GluA2). Spine density in 3–4 dendritic branchlets of Purkinje cells from wild-type or SCA1 mice 6 days after a single injection of vehicle or Ro0711401 is shown in **(B)**, where values are means + S.E.M. of 4 mice per group. The distribution of dendritic spines in relation to their lengths in distal dendrites of Purkinje cells 6 days after a single injection of vehicle or Ro0711401 in symptomatic SCA1 mice or after injection of vehicle in wild-type littermates is shown in **(C)**. The length of 200 spines per mouse was measured. Values are means ± S.E.M. of 5 individual determination for groups. In **(**C**)** spines were identified and measured using Image Pro Plus Software. Representative images and distribution spine analysis are shown. Data are means ± S.E.M. of 4 mice per group. *p < 0.05 (Two-Way ANOVA + Bonferroni’s t tests) as indicated in individual groups of columns. F_(13,84)_ = 87.47.

We reasoned that the long-lasting improvement in motor performance caused by a single injection of Ro0711401 could reflect the induction of adaptive mechanisms associated with activity-dependent synaptic plasticity and cerebellar motor learning. Changes in the expression, Ser-880 phosphorylation, and clustering of the Ca^2+^-impermeable GluA2 subunit of AMPA receptors, have been associated with the induction and expression of cerebellar LTD [32-37]. Levels of both unphosphorylated and Ser880-phosphorylated GluA2 subunit were found to be significantly elevated in the cerebellum of SCA1 mice 6 days following a single injection of Ro0711401 (Figure 8A). This treatment did not cause changes in the number of dendritic spines in Purkinje cells of SCA1 mice (Figure 8B). However, injection of Ro0711401 did cause changes in the *morphology* of dendritic spines that might be associated with motor learning. The distribution analysis of a fixed number of dendritic spines of Purkinje cells in relation to their length showed a shift to the right in symptomatic SCA1 mice as compared to age-matched wild-type mice. A single injection of Ro0711401 in SCA1 mice increased the number of “short” dendritic spines (i.e. spines with length between 0.3 and 0.5 μm), changing the pattern of distribution in a way similar to that found in wild-type mice (Figure 8C).

## Discussion

We confirmed here the reduced expression of mGlu1 receptors in the cerebellum of SCA1 mice (see Introduction and references therein). At least the reduction of the transcript of mGlu1 receptors was confirmed when values were normalized to calbindin mRNA levels, suggesting that receptor expression was reduced in individual Purkinje cells. Interestingly, Purkinje cells of symptomatic SCA1 mice showed an intense mGlu5 receptor immunoreactivity, which was nearly absent in Purkinje cells from control mice. In the cerebellum, the mGlu5 receptor is highly expressed early after birth, and then progressively disappears concomitantly with the appearance of mGlu1 receptors [9,38]. Our data raise the interesting possibility that in Purkinje cells mGlu1α and mGlu5 receptors are mutually exclusive and that the highly expressed mGlu1α receptor maintains the mGlu5 receptor repressed in the adult life. Accordingly, (i) the mGlu5 receptor reappeared in Purkinje cells of SCA1 mice, which were characterized by a reduced expression of mGlu1α receptors; (ii) amplification of the residual mGlu1α receptors (or other mGlu1 splice variants) with Ro0711401 repressed again the mGlu5 receptor in SCA1 mice; and (iii) pharmacological blockade of mGlu1 receptors with JNJ16269685 markedly enhanced the expression of mGlu5 receptors in Purkinje cells. A similar scenario was seen in mice subjected to experimental autoimmune encephalomyelitis (EAE) or in autoptic cerebellar samples from patients with multiple sclerosis, in which a reduced expression of mGlu1α receptors was associated with the reappearance of mGlu5 receptors in Purkinje cells [39]. mGlu1 and mGlu5 receptors are both coupled to phospholipase Cβ, the enzyme that cleaves PtdIns-4-5-P_2_ into InsP_3_ and DAG. However, the dynamics of InsP_3_-stimulated intracellular Ca^2+^ release differs between the two receptors, showing a peak in cytosolic Ca^2+^ followed by a plateau phase, and Ca^2+^ oscillations in response to mGlu1 and mGlu5 receptor activation, respectively [40]. Ca^2+^ oscillations are linked to basic processes of cell biology, such as cell proliferation, differentiation, and survival [41,42], whereas a single peaked Ca^2+^ response might be a feature of a mature excitable cell. Thus, the early expression of mGlu5 receptors may serve to support mechanisms of Purkinje cell maturation, which are override by the “synaptic” mGlu1 receptor when maturation is completed. The reappearance of mGlu5 receptors associated with the reduced expression (or function) of mGlu1α receptors might be viewed as a sterile attempt to compensate for the loss of mGlu1α receptors, or more simply a restatement of a developmental pattern of intracellular calcium mobilization associated with Purkinje cell pathology. Remarkably, treatment with the mGlu5 PAM, VU0360172, which was proven to be centrally active after systemic administration [43,44] caused only small and transient improvements in motor performance in SCA1 mice.

In contrast, pharmacological enhancement of the defective mGlu1 receptor caused a prolonged improvement of motor coordination in SCA1 mice. We used the selective mGlu1 receptor PAM, Ro0711401, which has nanomolar affinity for mGlu1 receptors, that is systemically active, and shows an elimination half-life < 2 hours [45]. Systemic injection of Ro0711401 was shown to reduce the frequency of spike-and-wave discharges in a rat model of absence epilepsy [46], and to improve motor signs in EAE mice [39]. Of note, treatment with Ro0711401 had no effect on motor behavior in control mice or in presymptomatic SCA1 mice and did not cause gross anatomical or behavioral abnormalities besides the improvement of motor performance in symptomatic SCA1 mice. Thus, Ro0711401 might be considered as a prototypical compound for the development of new symptomatic drugs in the treatment of SCA1 and perhaps other cerebellar disorders.

We were surprised to find that the motor improving effect of Ro0711401 lasted well beyond the time of drug clearance from the cerebellar tissue and was still seen 6 days following a single injection. This long-term effect of Ro0711401 was not associated with an up-regulation of mGlu1 receptors in Purkinje cells and could not be ascribed to the disappearance of mGlu5 receptors because treatment with VU0360172 caused a short-lasting *improvement* of motor performance. In addition, pharmacological inhibition of mGlu5 receptors has no effect on motor coordination in EAE mice, in which mGlu5 receptors are also up-regulated [39]. Ro0711401 could facilitate the induction of cerebellar LTD during the repeated execution of motor tasks at the rotarod, thereby inducing a form of procedural memory, which might be formed initially in the cerebellar cortex and then transferred to vestibular nuclei to be consolidated to a long-term memory [47]. During LTD induction, the glutamate released from parallel fibers activates perisynaptic mGlu1 receptors in the distal dendrites of Purkinje cells with ensuing mobilization of intracellular Ca^2+^ and activation of PKC. The conjunctive activation of climbing fibers provides an additional source of intracellular Ca^2+^ mediated by the opening of voltage-sensitive Ca^2+^ channels. PKC activation phosphorylates the GluA2 subunit of AMPA receptors leading to AMPA receptor internalization. The DAG generated by inositol phospholipid hydrolysis is also a metabolic source for the endocannabinoid, 2-arachidonylglycerol, which contributes to depress synaptic transmission by activating presynaptic CB1 receptors [32,48,49]. Chung et al. [33] have shown for the first time that phosphorylation of the GluA2 subunit, on Ser 880 within its C-terminal PSD-95/Disc Large/Zona Occludens-1 (PDZ) ligand, by PKCα is required for the induction of LTD. This phosphorylation promotes AMPA receptor endocytosis, a process that is tightly regulated by a number of GluA2-interacting proteins, such as protein interacting with C-kinase 1 (PICK1), N-ethylmaleimmide-sensitive factor (NSF), and glutamate receptor-interacting proteins (GRIP) 1 and 2 [34-36]. We found that Ro0711401 treatment in SCA1 mice enhanced Ser 880 phosphorylation of the GluA2 subunit within the context of increased total GluA2 levels. This suggests that pharmacological enhancement of mGlu1 receptors enriches AMPA receptors of the Ca^2+^-impermeable GluA2 subunit, and that phosphorylation of GluA2 subunit on Ser 880 commits AMPA receptors to endocytosis. Another finding that is suggestive of an LTD-promoting activity of mGlu1 receptor enhancement is the different distribution profile of dendritic spine length in Purkinje cells between SCA1 mice treated with vehicle and those treated with Ro0711401. Recent studies suggest that genetic manipulations causing defective LTD of the parallel fiber-Purkinje cell synapses and impairment of motor learning and motor coordination lead to an increased number and length of dendritic spines in Purkinje cells [50,51]. We analyzed a fixed number of dendritic spines per mouse, and, therefore, we could not determine the real number of spines in distal dendrites in our groups of mice. However, we found a higher proportion of long dendritic spines (0.7-1 μm) in symptomatic SCA1 mice treated with vehicle with respect to wild-type littermates. In contrast, SCA1 mice treated with Ro0711401 showed a higher proportion of short dendritic spines (0.3-0.5 μm) similarly to what found in wild-type mice. However, we wish to highlight that the relationship between the number and shape of dendritic spines in Purkinje cells and cerebellar LTD is debated, and two-photon microscopy analysis failed to detected changes in dendritic spines after synaptic or chemical LTD at the parallel fiber-Purkinje cell synapses, and manipulations that caused persistent retractions of dendritic spines did not alter parallel fiber-evoked postsynaptic currents [52].

## Conclusions

The prolonged motor improvement induced by Ro0711401 strongly encourages the development of mGlu1 receptor PAMs for the treatment of SCA1. There is currently no cure for this disorder and there is no way of slowing its progression. Some associated symptoms, such as extrapyramidal motor symptom, anxiety and sleep disturbances can be treated medically, but, to our knowledge, there are no drugs that specifically target the defect in cerebellar motor programming underlying ataxia and other cerebellar symptoms associated with SCA1. Putative “cerebellomodulatory” drugs, such as buspirone, zolpidem, riluzole, and amantadine (all of these have indications for other disorders) were found to have no effect on motor performance in SCA1 mutant mice [53]. Beneficial effects on motor symptoms and Purkinje cell degeneration were found with the potassium channel blocker, 3,4-diaminopyridine [54]. This drug is used for the treatment of Lambert-Eaton myasthenic syndrome with an acceptable profile of safety and tolerability, although epileptic seizures and arrhythmias are experienced in response to 3,4-diaminopyridine as a result of the widespread expression of type-A potassium channel K_v_4.3 in excitable cells [55]. Lithium treatment can also improve motor symptoms in SCA1 mice by apparently targeting pyramidal neurons in the hippocampus [56]. Lithium treatment, however, can induce long-lasting neurological sequelae, the most frequent of which is a permanent cerebellar syndrome characterized by Purkinje cells degeneration [57].

mGlu1 receptor PAMs are excellent candidates as symptomatic drugs in SCA1 for the following reasons: (i) expression of mGlu1 receptors is prominent in cerebellar Purkinje cells, and these receptors play a key role in mechanisms of activity-dependent synaptic plasticity underlying motor learning; (ii) mGlu1 receptors are directly linked to the pathophysiology of SCA1 (see Introduction and references therein); (iii) all mGlu receptor ligands tested in clinical trials have shown, in general, a good profile of safety and tolerability [5]; (iv) a clear advantage of a PAM is that this type of drug acts in an activity-dependent fashion by discriminating receptors that are recruited into function by the endogenous glutamate with respect to those that are not active (see above); and (v) as opposed to other receptor subtypes, mGlu1 receptors have no recognized role in peripheral organs [58], with the exception of melanoma cells in which these receptors are ectopically expressed [59,60].

Thus, our findings may lay the groundwork for the design of cerebellum-specific, effective, and safe symptomatic drugs for the treatment of SCA1 and perhaps other ataxic disorders.

## Methods

### Materials

The selective positive allosteric modulator of mGlu1 receptor Ro0711401 [9H-xanthene-9-carboxylic acid(trifluomethyl-oxazol-2-yl)amide] was kindly provided by La Roche Ltd, Pharmaceutical Division, Basel, Switzerland. JNJ16259685 (3,4-dihydro-2H-pyrano[2,3-b]quinolin-7-yl)-(cis-4-methoxycyclohexyl)-methanone and VU0360172 (N-cyclobutyl-6-[2-(3-fluorophenyl) ethynyl]-3-pyridinecarboxamide hydrochloride) were purchased from Tocris Cookson (Avonmouth, Bristol, UK). All other chemicals products were purchased from Sigma-Aldrich (S. Louis, MO).

### Animals

Heterozygous B6.129S-Atxn1tm1Hzo/J mice (stock number 005601) were obtained from The Jackson Laboratory (Bar Harbor, ME). Wild-type littermates were generated from the colony. mGlu1 receptor-deficient *crv4* mice and their wild-type counterparts were provided by A.M.P. (Gaslini Institute, Genoa, Italy). The *crv4* mutation is a spontaneous recessive mutation consisting of a retrotransposon long terminal repeat fragment insertion that disrupts the splicing of the mGlu1 receptor gene (Grm1) and causes the absence of the protein [17]. Mice were kept under environmentally controlled conditions (ambient temperature, 22°C; humidity, 40%) on a 12 h light/dark cycle with food and water *ad libitum*. All experiments were carried out according to the European (86/609/EEC) and Italian (D:Lgs. 116/92) guidelines of animal care. The experimental protocol was approved by the Italian Ministry of Health (D.M.209/2011-B). All efforts were made to minimize animal suffering and the number of animals used. SCA1 mice were studied at 4 and 30 week of age and compared with wild-type littermates. Eight week old *crv4* mice and their wild-type counterparts were used as reference mice to exclude off-target effects of Ro0711401.

### Experimental design

Different groups of pre-symptomatic SCA1 mice (4-week old), symptomatic SCA1 mice (30-week old) and their wild-type littermates were used for the analysis of mGlu1 and mGlu5 receptor expression in the cerebellum. Additional groups of symptomatic SCA1 mice and wild-type littermates were treated systemically with one of the following compounds: the mGlu1 receptor positive allosteric modulator (PAM), Ro0711401 (10 mg/kg, s.c.), the mGlu1 receptor negative allosteric modulator (NAM), JNJ16259685 (2.5 mg/kg, i.p.), or the mGlu5 receptor PAM, VU0360172 (10 mg/kg, s.c.). Ro0711401 was dissolved in peanut oil; JNJ16259685 was dissolved in saline containing 10% hydroxypropyl-β-cyclodextrin; VU0360172 was dissolved in 10% Tween 80. Control mice were treated with the respective vehicles s.c. or i.p. Some groups of mice (typically 4–6 mice for groups) received a single injection of each of the three drugs, and motor performance was assessed at different post injection times (from 30 min to 6 days). Independent groups of symptomatic SCA1 mice received daily injection of vehicle or Ro0711401, and motor behavior was assessed 1 hour after a single injection. The same groups of SCA1 mice and wild-type littermates acutely treated with vehicle or Ro0711401 which were used for the assessment of motor behavior up to 6 day post-injection were also used for measurement of mGlu1 and mGlu5 receptors, GluA2 subunit of AMPA receptor and the length of dendritic spines in Purkinje cells. One independent group of SCA1 mice was used for measurements of Ro0711401 levels in the cerebellum after a single drug injection. To test the specificity of mGlu1 receptor PAM, we examined motor performance 1 hour after a single injection of Ro0711401 in mice lacking mGlu1 receptor (*crv4*) and their wild-type littermates. Finally, independent groups of wild-type mice were used to examine the effect of single and repeated injections of JNJ16259685 on mGlu5 receptor expression in the cerebellum. In these particular experiments, a first group of wild-type mice received daily injections of vehicle for 6 days. A second group of mice received daily injections of vehicle for 5 days followed by a single injection of JNJ16259685. A third group received daily injections of JNJ16259685 for 5 days.

### Real-time RT-PCR

Total RNA from the cerebellum was isolated using the TRIzol reagent (Life Technologies, Monza, Italy) according to the manufacturer’s protocol and retrotranscribed into cDNA by using SuperScript III Reverse Transcriptase (Life Technologies). Real-Time RT-PCR was performed on the Step One Plus Applied Biosystems. PCR was performed by using Power SYBR Green PCR Master Mix Kit (Life Technologies) according to the manufacturer’s instructions. Real-time PCR was performed by using the following primers:

mGlu1α/β receptors: forward, 5′-CATACGGAAAGGGGAAGTGA-3′; reverse, 5′-AAAAGGCGATGGCTATGATG-3′. mGlu5 receptors: forward, 5′-ACGAAGACCAACCGTATTGC-3′; reverse, 5′-AGACTTCTCGGATGCTTGGA-3′. Calbindin: forward, 5′-GGAGCTATCACCGGAAATGA-3′; reverse, 5′-AGTTGCTGGCATCGAAAGAG-3′. GAPDH: forward, 5′-CGTCCCGTAGACAAAATGGT-3′; reverse, 5′-TCAATGAAGGGGTCGTTGAT-3′. mRNA levels were calculated from serially diluted standard curves simultaneously amplified with the samples and normalized to calbindin and GAPDH mRNA levels.

### Western blot analysis

The cerebella were dissected out and homogenized at 4°C in Tris–HCl buffer containing 1 mM PMSF, pH 7.4, and an aliquot was used for protein determinations. Equal amounts of proteins (30 μg) from supernatants were separated by 8% SDS polyacrilamide gel for the detection of mGlu1α receptors, mGlu5 receptors, GluA2 AMPA receptor subunit, and (Ser880)phosphorylated-GluA2 subunit using a mini-gel apparatus (Bio-Rad Mini Protean II cell). Proteins were than electroblotted on Immuno PVDF membranes (Bio-Rad, Milano, Italy) for 1 hour using a semi-dry electroblotting system (Bio-Rad). Filters were washed three times and blocked for 1 hour in Tris-Tween buffered saline (TTBS) containing 5% non-fat dry milk. The following primary antibodies were used: rabbit polyclonal anti-mGlu1α antibody (1 μl/ml, Upstate Biotechnology, Lake Placid, NY); rabbit polyclonal anti-mGlu5 antibody (1 μl/ml, Upstate Biotechnology); rabbit polyclonal anti-GluA2 (1 μl/ml, Upstate Biotechnology) antibody; rabbit polyclonal anti-phospho-GluA2 (Ser880) (1:500, Upstate Biotechnology) antibody. Filters were washed three times with TTBS buffer and then incubated for 1 hour with secondary peroxidise-coupled anti-rabbit antibodies (anti-rabbit 1:7000 Calbiochem, Milano, Italy). Immunostaining was revealed by enhanced chemiluminescence luminosity (Amersham Pharmacia Biotech, Arlington Height, IL). The blots were re-probed with monoclonal anti β-actin antibody (1:250, Sigma, St. Louis, MO).

### Immunohistochemistry

For cerebellum paraffined slices, serial sections were deparaffinized and soaked in 3% hydrogen peroxide to block endogenous peroxidase activity, incubated overnight with anti-mGlu1α or mGlu5 receptor antibodies (both at 1:100) or with mouse polyclonal anti-calbindin antibody (1:500; Abcam, Cambridge, UK), and then incubated for 1 hour with secondary biotinylated anti-rabbit antibodies (1:200; Vector Laboratories, Burlingame, CA). Control staining was performed without primary antibodies. The immunoreaction was carried out with 3,3-diaminobenzidine tetrachloride (ABC Elite kit; Vector Laboratories).

For frozen serial sections, the cerebellum was removed, post-fixed overnight in 4% PFA, placed in 30% sucrose/0.1 M phosphate buffer for 24 hours and cryosectioned. Slices were stained with primary anti-mGlu1α receptor, anti-mGlu5 receptor, anti-calbindin, followed by the appropriate fluorescent secondary antibodies. All images were captured using a Zeiss Carl Axiophot2 microscopy (Zeiss, Gottingen, Germany) and processed with NIS-elements F3.0.

### Confocal microscopy

Mice were perfused with ice-cold 4% paraformaldehyde, and equilibrate with 30% sucrose overnight. Cerebellum was sectioned using a Leica cryostat (CM3050). For immunofluorescence analysis, serial sections were incubated with blocking solution (5% normal serum in 0.30% Triton X-100 in PBS) and then with the following antibodies overnight at 4°C: anti-calbindin (1:500, Abcam), anti-vGluT2 (1:250, Millipore, Temecula, CA), anti-mGlu5 receptor (1:200, Abcam). After washing, sections were incubated with secondary antibodies conjugated with Alexa Fluor 488 (1:200, Invitrogen) and Cy3 (1:200, Jackson immunoResearch, Suffolk, UK) for 2 hours at room temperature and rinsed in PBS. Finally sections were mounted with anti-fading agent (Vector) and examined with Olympus FV1000 spectral confocal laser scanning microscope.

### Golgi staining

Five mice per group were deeply anesthetized with 320 mg/kg of chloral hydrate and perfused with 0.9% saline. Brains were removed and placed in 20 ml Golgi-Cox solution [61]. The brains were stored in the dark for 3 weeks and refreshing solutions every 2–3 days, after which the Golgi-Cox solution was replaced with 30% sucrose. The brains were allowed to sit in the dark for 2–5 days before sectioning at vibratome and processing for the staining in according with Gibb and Kolb [62]. Distal, third or fourth order dendrites, were used for the evaluation of spine length, defining a spine as a protrusion clearly originating from the shaft of a dendrite and not intersecting any other dendritic segment; moreover, we did not measure any spine that appears to contain more than one head [50]. Spines were identified and measured using a EC-Plan Neofluar 100×, 1,3 NA oil immersion objective (Zeiss). Measurement was performed using Image Pro Plus Software (Media Cybernetics, Rockville, MD). We captured images of ten cells for animal, and for each cell, we measured the length in twenty spines, for a total of two-hundred spines measured for animal. The corresponding values from ten cells per animal were averaged, and the average of the five animal per group was used for statistical analysis. Statistical analysis were performed using Two-Way ANOVA analysis of variance, followed by Bonferroni’s post tests.

### Measurement of spine density

Dendritic protrusions were quantified in terms of dendritic spines density. These were counted in 50 μm from 3 to 4 dendritic branchlets of cerebellar Purkinje cells. Since distal dendritic branchlets measure often less than 50 μm in length, more than one segment per cell were selected until reaching the measure established of 50 μm. These segments were selected randomly. Counting was performed by direct observation in ten cells per animal. Statistical analysis were performed using One-Way ANOVA analysis of variance, followed by Bonferroni post test.

### HPLC-MS/MS analysis

#### *Sample preparation*

The cerebellum was homogenated with 1 ml of 0.1% aqueous formic acid. For each sample, weight was recorded. Thirty μl of tissue homogenates were added to 150 μl of internal standard working solution (1 mM dansilnorvaline in 100% acetonitrile). After extensive vortex (60 sec), samples were centrifuged at 14,000 revolutions per minute (rpm) for 5 min. Forty μl of supernatant were mixed with 160 μl of 0.1% aqueous formic acid and transferred to an autosampler vial for injection into the chromatographic system.

#### *Chromatographic conditions*

The HPLC analysis was performed using an Agilent Liquid Chromatography System series 1100 (Agilent Technologies, Santa Clara, CA), with a binary pump, an autosampler, a solvent degasser and a column oven. The chromatographic separation was performed using a 50 × 2.0 mm, Luna C18, 5 μm, 100 Å pore size column (Phenomenex, Torrance, CA), equipped with Security guard precolumn (Phenomenex), containing the same packing material. The column was maintained at room temperature. The mobile phase was a solution of 0.1% aqueous formic acid (eluent A) and 100% acetonitrile (eluent B). Injection volume consisted of 20 μl; elution was performed at flow rate of 300 μl/min, using 10% solvent B for 1 min, linear gradient to 90% solvent B for 3 min, 90% solvent B for 2 min and afterwards re-equilibrating with 90% solvent A for 4 min. The total analysis run time was 10 min.

#### *Mass spectrometry conditions*

The mass spectrometry was performed on a 3200 triple quadrupole system (Applied Biosystems, Foster City, CA), equipped with a Turbo Ion Spray source. Data were acquired and processed with Analyst 1.4.2 software. The detector was set in the positive ion mode. The ion spray voltage was set at 5000 V and the source temperature was 300°C. The collision activation dissociation (CAD) gas was set at medium value and nitrogen was used as collision gas. The Q1 and Q3 quadrupoles were tuned for the unit mass resolution. The transitions of the precursor ions to the product ions were monitored with a dwell time of 100 ms for each analyte. The instrument was set in the multiple reaction monitoring (MRM) mode. Mass spectrometer parameters were optimized to maximize sensitivity for all transitions (Table 2).

**Table 2:** Monitored ion transitions and their parameter settings of mass spectrometer for the detection and measurements of Ro0711401

**Precursor ion**	**Fragment**	**DP**	**EP**	**CE**	**CXP**
**( *m/z *, amu)**	**( *m/z *, amu)**	**(V)**	**(V)**	**(V)**	**(V)**
**RO (361.0)**	181.1	47.0	5.8	25.0	2.9
	153.0	47.0	5.8	19.1	2.8
**IS (351.2)**	234.0	42.0	7.0	23.7	3.2
	170.0	42.0	7.0	26	2.6

DP – Declustering Potential; EP – Entrance Potential; CE – Collision Energy; CXP – Collision Cell Exit Potential.

A calibration curve was established using known concentration (0, 6.25, 12.5, 25, 50, 100, 250, 500, 1000 ng/ml) of Ro0711401 dissolved in 0,1% aqueous formic acid and processed in the same way of tissue samples. The equation of linear regression obtained for this value range was y = 0.000204× + 0.0033 (r = 0.9982).

### Assessment of motor coordination in SCA1 mice

#### *Rotarod test*

Motor ability was assessed using an accelerating Rotarod apparatus, as described [63]. Mice were forced to manage an accelerating rotarod to monitor motor performance. The rotarod apparatus consisted of a rotating horizontal cylinder (30 mm) and a motor driver control unit (Ugo Basile, Varese, Italy). The cylinder was divided into 5 separate rotating compartments, fully enclosed to ensure that the mice did not jump out of their area. The mice were placed on the rod which was rotating at accelerating speed from 5 to 15 rpm. Automatic timers recorded the duration of time the mice remained on the rod, and two infrared beams at the base of each compartment determined when the mice had fallen off the rod. Mice were tested using four trials per day (10 min rests between trials) on 4 consecutive days. During a trial, the rod accelerated from 4 to 40 rpm over 5 min. Each trial lasted until the mouse fell from the rod or for a maximum of 5 min.

#### *Paw printing test*

Paw prints were made with ink and water color paper. Ink was applied to the hind paws of individual mice which were then induced to walk forward, leaving a record on the paper [15]. The test was carried out twice soon after the Rotarod test. Data were quantified by calculating the coefficient of variation (the ratio between standard deviation and the mean value) of five consecutive strides with the left paw.

### Statistical analysis

Statistical analysis were carried out by using the Student’s t test (Figures 1, 2, 8); paired t-test (Figure 3); One-Way ANOVA (Figures 3, 6) or Two-Way ANOVA for repeated measures followed by Fisher’s LSD (Figures 3, 4, 5) or followed Bonferroni’s t-test (Figure 8); p values < 0.05 were considered significant.

## Abbreviations

mGlu: Metabotropic glutamate; SCA1: Spinocerebellar ataxia type-1; PAM: Positive allosteric modulator; NAM: Negative allosteric modulator; ATXN1: Ataxin-1; InsP3: Inositol-1,4,5-trisphosphate; DAG: Diacylglicerol; PKC: Protein kinase C; AMPA: 2-amino-3-(3-hydroxy-5-methyl-isoxazol-4-yl)propanoic acid; LTD: Long term depression.

## Competing interests

The authors declare that they have no competing interests.

## Authors’ contributions

SN, CZ and FF designed and performed *in vivo* experiments and analyzed data. MC and PS performed real-time PCR experiments. FB, DB and FW performed immunohistochemical analysis. GM and MS performed confocal microscopy analysis.LL and MS analyzed sample by HPLC. RG and GB contributed to experiment design and supervised research. FN designed experiments, supervised research and wrote the manuscript. All authors read and approved the final manuscript.
